# Antitumor activity of the c-Myc inhibitor KSI-3716 in gemcitabine-resistant bladder cancer

**DOI:** 10.18632/oncotarget.1545

**Published:** 2014-01-16

**Authors:** Ho Kyung Seo, Kyung-Ohk Ahn, Nae-Rae Jung, Ji-Sun Shin, Weon Seo Park, Kang Hyun Lee, Sang-Jin Lee, Kyung-Chae Jeong

**Affiliations:** ^1^ Center for Prostate Cancer, Hospital, National Cancer Center, Goyang, Gyeonggi-do, Korea; ^2^ Biomolecular Function Research Branch, Research Institute, National Cancer Center, Goyang, Gyeonggi-do, Korea; ^3^ Genitourinary Cancer Branch, Research Institute, National Cancer Center, Goyang, Gyeonggi-do, Korea

**Keywords:** Bladder cancer, c-Myc, inhibitor, gemcitabine, gemcitabine resistance

## Abstract

Intravesical instillation of chemotherapeutic agents is a well-established treatment strategy to decrease recurrence following transurethral resection in non-muscle invasive bladder cancer. Gemcitabine is a recently developed treatment option. However, the curative effects of gemcitabine are far from satisfactory due to *de novo* or acquired drug resistance. In a previous study, we reported that intravesical administration of the c-Myc inhibitor KSI-3716 suppresses tumor growth in an orthotopic bladder cancer model. Here, we explored whether KSI-3716 inhibits gemcitabine-resistant bladder cancer cell proliferation. As expected from the *in vitro* cytotoxicity of gemcitabine in several bladder cancer cell lines, gemcitabine effectively suppressed the growth of KU19-19 xenografts in nude mice, although all mice relapsed later. Long-term *in vitro* exposure to gemcitabine induced gemcitabine-specific resistance. Gemcitabine-resistant cells, termed KU19-19/GEM, formed xenograft tumors even in the presence of 2 mg/kg gemcitabine. Interestingly, KU19-19/GEM cells up-regulated c-Myc expression in the presence of the gemcitabine and resisted to the gemcitabine, however was suppressed by the KSI-3716. The sequential addition of gemcitabine and KSI-3716 inhibited gemcitabine-resistant cell proliferation to a great extent than each drug alone. These results suggest that sequential treatment with gemcitabine and KSI-3716 may be beneficial to bladder cancer patients.

## INTRODUCTION

Bladder cancer is the sixth most commonly diagnosed cancer in the United States [1]. Approximately 80% of patients with bladder cancer present with non-muscle invasive bladder cancer (NMIBC) initially [2, 3], Typically, patients are treated with a complete transurethral resection of the tumor followed by intravesical instillation of antitumor agents. Bacillus Calmette-Guerin (BCG) is the most effective adjuvant agent for treating NMIBC [4]. Nonetheless, approximately 30–40% of NMIBC patients do not respond to BCG treatment; of the initial responders, 35% relapse within 5 years [5]. Intravesical instillation of gemcitabine is effective against BCG-refractory NMIBC as well as advanced bladder cancer [5–8]. Several randomized trials of intravesical gemcitabine therapy demonstrate that tumor recurrence is 25–53.1% in BCG-refractory NMIBC [9].

Gemcitabine (2′,2′-difluorodeoxycytidine) is a synthetic pyrimidine nucleoside analogue that has structural and metabolic similarities to deoxycytidine and cytosine arabinoside [10]. Its active metabolites are incorporated into DNA and inhibit DNA polymerase thereby inhibiting DNA synthesis and inducing apoptosis [11]. Intravenous administration of gemcitabine is highly effective and well tolerated; therefore, this agent is used as both a first- and second-line chemotherapy, in combination or as a single agent, for the treatment of metastatic bladder cancer [8]. A combination of gemcitabine and cisplatin is considered the standard therapy for patients with locally advanced and metastatic bladder cancer. However, despite reasonable response rates to initial chemotherapy in patients with metastatic bladder cancer, long-term disease-free survival rates remain disappointing. Gemcitabine has shown single-agent response rates of 28–36% in previously untreated metastatic bladder patients, with mild myelosuppression [12].

This limited efficacy may be due to *de novo* drug resistance and/or the development of a drug-resistant cellular phenotype during treatment. Drug resistance can be acquired at the genetic level through gene amplification, the transcriptional level through epigenetic modifications, or the proteomic level through mutation or aberrant expression. Gemcitabine is predominantly transported into the cell by human equilibrative and concentrative nucleoside transporters (hENT and hCNT, respectively). Cells deficient in hENTl are highly resistant to gemcitabine [13]. As a prodrug, gemcitabine is phosphorylated to produce its active diphosphate and triphosphate metabolites, which inhibit ribonucleotide reductase (RR) and DNA synthesis, respectively. Deoxycytidine kinase (dCK) is the rate-limiting enzyme in the biotransformation of nucleoside analogs and increases in dCK activity may improve the efficacy of gemcitabine [14]. Furthermore, increased expression of the catabolic enzymes 5′-nucleotidase (5′-NT) and cytidine deaminase (CDA) has been found in many cell lines resistant to gemcitabine [15, 16]. Finally, non-small cell lung cancer patients with low level expression of the Ml subunit of RR (RRM1) significantly benefited from gemcitabine/cisplatin neoadjuvant chemotherapy [17], while resistance to gemcitabine was observed in cells overexpressing both RRM1 and RRM2 [18, 19]. Additionally, faulty processing of microRNA (miRNA) coding genes, and consequent altered function of the miRNA, can also result in drug resistance. For example, in bladder cancer cell lines miRNAs 1290, 138, let-7i, and let-7b impart resistance to gemcitabine in part through the modulation of mucin-4 [20].

Many studies have highlighted the important role of c-Myc in the development of drug-resistant phenotypes in cancer [21, 22]. For instance, it has been reported that in human breast epithelial cells, c-Myc overexpression is coupled to the modulation of drug transporter gene expression [23], and c-Myc inhibition also sensitizes Lewis lung carcinoma to cisplatin, taxol, and etoposide. Interestingly, cyclical administration of cisplatin and *c-myc* antisense oligomers was more potent than co-administration [24]. However, it is not known if the development of gemcitabine resistance is associated with c-Myc overexpression in bladder cancer. Therefore, in the present study, we initially developed a gemcitabine-resistant human bladder cancer cell line by continuous exposure to gradually increasing, clinically relevant doses of gemcitabine. We then addressed the functional role of c-Myc during the development of gemcitabine resistance and further investigated the efficacy of a c-Myc inhibitor against gemcitabine-resistant bladder cancer cells.

## RESULTS

### Gemcitabine is cytotoxic to various bladder cancer cell lines

**Figure 1:**
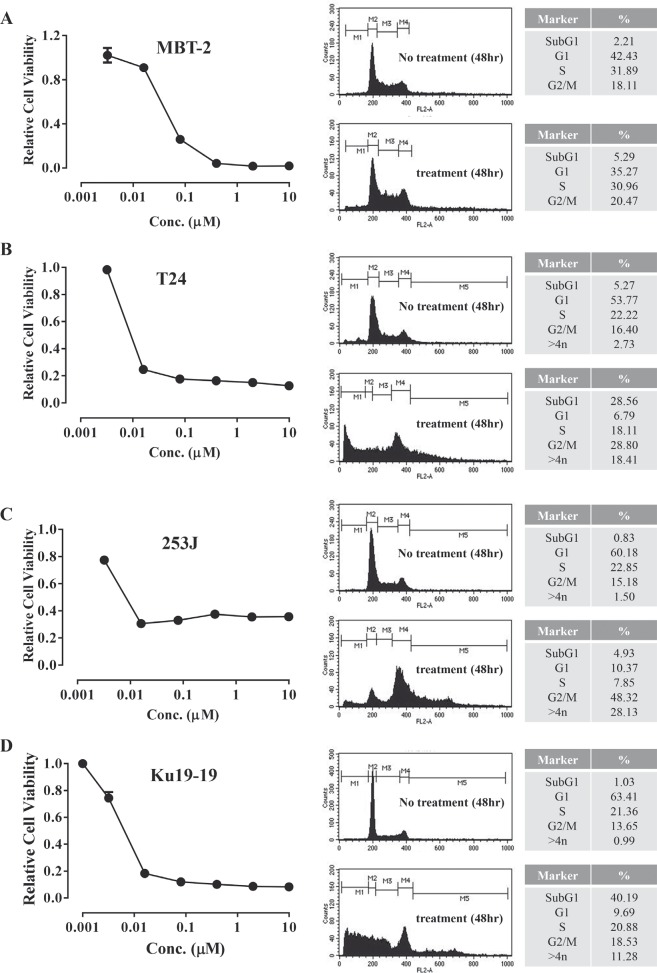
Gemcitabine inhibits proliferation of bladder cancer cells Cells (3 × 10^3^ cells/well) were plated in 96-well plates and treated with various concentrations of gemcitabine (0–10 μM) for 72 hrs. Cell proliferation assays were performed by counting the number of viable cells. For cell cycle analysis, 2 × 10^5^ cells were plated in 100 mm dishes and treated with 1 μM gemcitabine for 48 hrs. Cell cycle phase was determined by flow cytometry.

Gemcitabine is already recognized as one of the most effective chemotherapeutic agents against bladder cancer. Here, we confirmed that gemcitabine effectively inhibits the proliferation of various bladder cancer cell lines and then determined the dose required to block bladder cell proliferation. Each bladder cell line was exposed to various concentrations of gemcitabine. Following incubation for 72 hrs, cell survival was determined by a cell viability assay. When cells were incubated for 72 hrs, gemcitabine inhibited cell survival by more than 70% at 0.1 μM in all cell lines tested. In most cell lines, a small number of cells survived doses as high as 10 μM gemcitabine. We hypothesized that the observed inhibition of survival resulted from cell cycle arrest and consequent apoptosis. Thus, cell cycle progression and apoptosis were analyzed. As expected, as gemcitabine is known to block DNA synthesis, flow cytometer analysis showed that a large proportion of cells in both the KU19-19 (40.19%) and T24 (28.56%) cell lines were in an apoptotic state. The proportion of cells undergoing apoptosis in the 253J and MBT-2 mouse bladder cancer cell line was relatively small (5.29% and 4.93%, respectively), although a large fraction of cells were arrested in the G2/M phase (Figure 1).

### In vivo tumors develop resistance to gemcitabine

**Figure 2:**
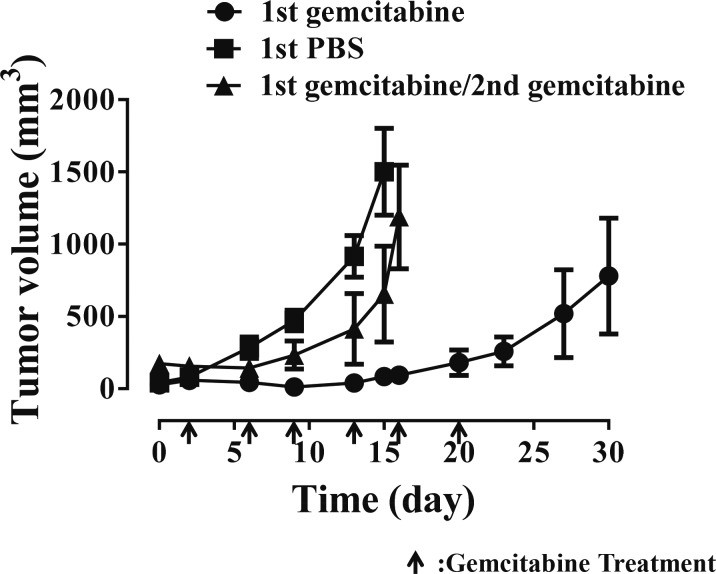
*In vivo* KU19-19 tumor xenografts develop resistance to gemcitabine Female BALB/c mice (6 weeks old) were subcutaneously inoculated with 2 × 10^6^ KU19-19 cells. Mice were intraperitoneally injected with 2 mg/kg of gemcitabine (●, 1st gemcitabine group) or PBS (■, control) twice a week for 3 weeks. Tumor growth was measured and volume calculated according to the following formula: length × (width)2 × 0.5236. At day 30, tumors from gemcitabine-treated mice were harvested and implanted in new BALB/c mice (6 weeks old). Mice were then treated with 2 mg/kg of gemcitabine twice a week for 3 weeks again (▲, 2nd gemcitabine group). Data represent the mean ± SEM. Statistical analysis was by unpaired *t*-test.

Next, gemcitabine-mediated *in vivo* tumor growth inhibition was evaluated. KU19-19 xenografts were established in BALB/c nude mice and mice were intraperitoneally administered gemcitabine at a dose of 2 mg/kg twice a week for 3 weeks (Figure 2). Generally, gemcitabine potently suppressed tumor growth provided that treatment was administered for at least six cycles. However, tumor growth was occasionally observed even after six treatment cycles, although growth rates were lower than in the control group (Figure 2, ■). These results indicate that gemcitabine slowed tumor growth rather than eliminating tumors. To determine whether tumors became resistant to gemcitabine or could be further suppressed with longer gemcitabine treatment, tumors were extracted, implanted into other BALB/c nude mice, and exposed to 2 mg/kg gemcitabine via intraperitoneal injection. Interestingly, these tumors (▲, 2nd gemcitabine group) grew as fast as the tumors in the control group (■), suggesting that the initial tumors (●, 1st gemcitabine group) were already resistant to gemcitabine. Gemcitabine resistance was not dependent on tumor size and drug penetration; instead, it is likely that a population of tumor cells acquired resistance or was inherently resistant to gemcitabine.

### Establishment of gemcitabine-resistant KU19-19 cells

**Figure 3:**
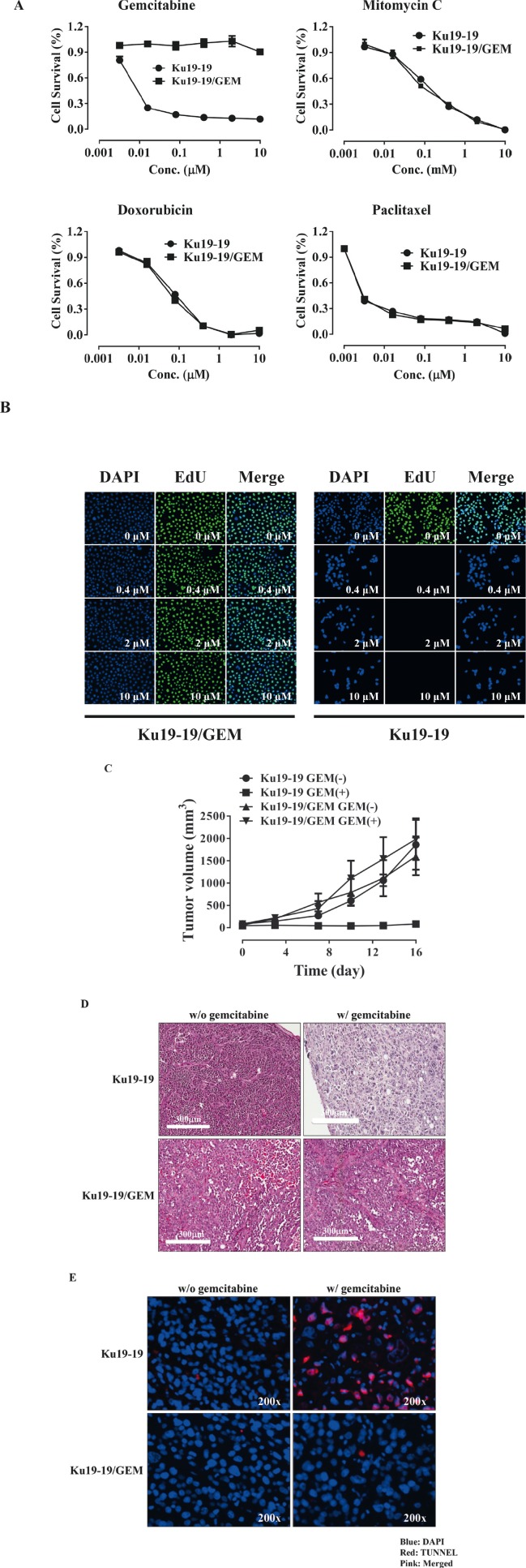
Development of a gemcitabine-resistant KU19-19 cell line KU19-19 cells were cultured in the presence of stepwise increasing concentrations of gemcitabine. Resistant cells, termed KU19-19/GEM, were obtained and continuously maintained in the presence of the same concentration of gemcitabine. (**A**) KU19-19/GEM or KU19-19 cells were plated in 96-well plates (3 × 10^3^ cells/well) and cell proliferation was measured after incubation with 0–10 μM of various chemotherapeutic reagents for 72 hrs. (**B**) KU19-19/GEM or KU19-19 cells were seeded in 96-well black plates (1 × 10^4^ cells/well) and treated with 0–10 μM gemcitabine. After incubation for 24 hrs, intercalated EdU was detected by fluorescence microscopy using 5 μM Alexa Fluor 488-conjugated azide. DAPI was used to stain cell nuclei.

After observing that a number of cells survived treatment with 0.1 μM gemcitabine to become the dominant culture in a tissue culture plate, we developed a gemcitabine-resistant cell line, termed KU19-19/GEM, by continuous exposure of KU 19-19 cells to step-wise increasing concentrations of gemcitabine (0.1–10 μM). TO evaluate whether KU19-19/GEM cells were selectively resistant to gemcitabine, KU19-19/GEM cells were exposed to a number of other drugs. As shown in Figure 3A, gemcitabine had no effect on KU19-19/GEM cells, whereas all other chemotherapeutic agents tested markedly reduced KU19-19/GEM (and KU19-19) cell proliferation, suggesting that KU19-19/GEM cells are selectively resistant to gemcitabine. Microarray data for KU19-19 and KU19-19/GEM cells also revealed several genes that were associated with cancer stem cell-like phenotypes of drug resistance (data not shown). Next, the effect of high doses of gemcitabine (up to 10 μM) on KU19-19/GEM cell proliferation was examined. The intercalating reagent EdU was continuously incorporated into KU19-19/GEM cells during DNA replication, in contrast to KU19-19 cells where little or no incorporation was observed, indicating that even high doses of gemcitabine fail to prevent KU19-19/GEM cell proliferation (Figure 3B). Next, we questioned whether KU19-19/GEM cells could still form tumors in mice. KU19-19/GEM cells (2 × 10^6^) were injected into the flank of BALB/c nude mice and tumor growth was monitored in the presence or absence of gemcitabine (Figure 3C). As shown, KU19-19/GEM cells retained the ability to form tumors, like the parental cell line KU19-19. As expected from the *in vitro* data, gemcitabine had no effect on KU19-19/GEM-derived tumor growth. Tumor sections harvested from mice were analyzed by hematoxylin and eosin staining, and TUNEL assays to observe apoptotic cells. As shown in Figure 3D and 3E, KU19-19/GEM cells were morphologically different from KU19-19 cells, and tumors from KU19-19 exhibited extensive apoptotic areas in the presence of gemcitabine.

(**C**) Female BALB/c mice were inoculated subcutaneously with 1 × 10^6^ KU19-19 or KU19-19/GEM cells. Mice were intraperitoneally injected with 2 mg/kg of gemcitabine twice a week for 3 weeks. Tumor growth was measured and plotted. Data represent the mean ± SEM. Statistical analysis was by unpaired *t*-test. (**D**) Tumors were harvested and histologically analyzed by hematoxylin and eosin staining. (**E**) TUNEL assays were performed on sections obtained from each tumor and cyanine 3-labeled TUNEL-positive cells were imaged under a fluorescence microscope.

### The c-Myc inhibitor KSI-3716 effectively induces KU19-19/GEM cell death

**Figure 4:**
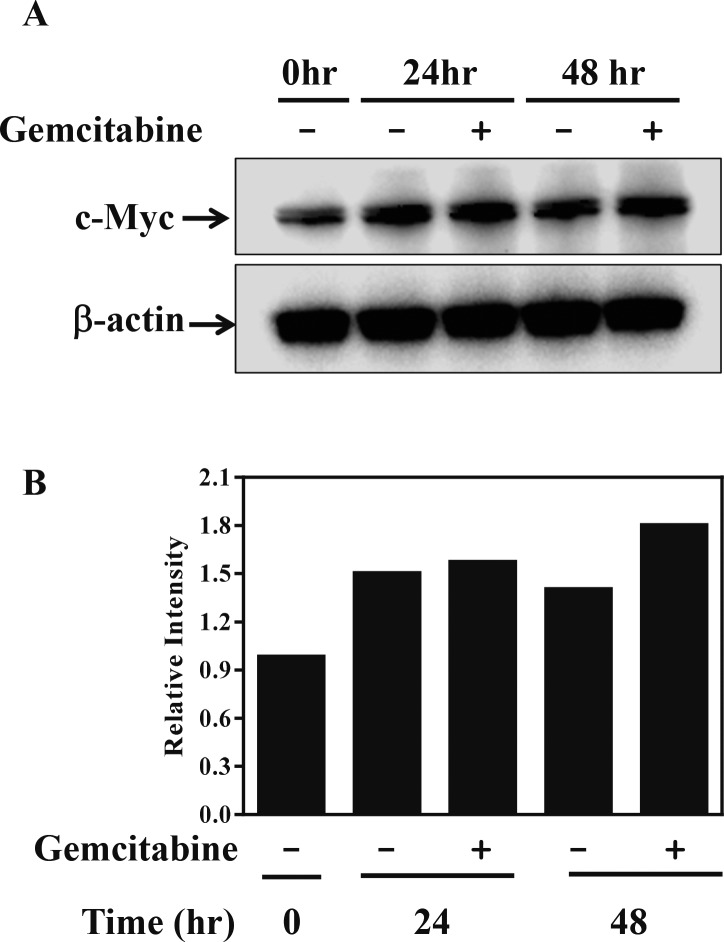
c-Myc expression is up-regulated in the presence of gemcitabine in KU19-19/GEM cells (**A**) KU19-19/GEM cells were treated with 10 μM gemcitabine for the indicated time, then lysed in RIPA buffer. Immunoblot analysis was performed using a total of 20 μg protein per lane, and c-Myc expression confirmed using a c-Myc antibody. (**B**) Proteins visualized by western blot in A were quantitated by densitometric analysis.

**Figure 5:**
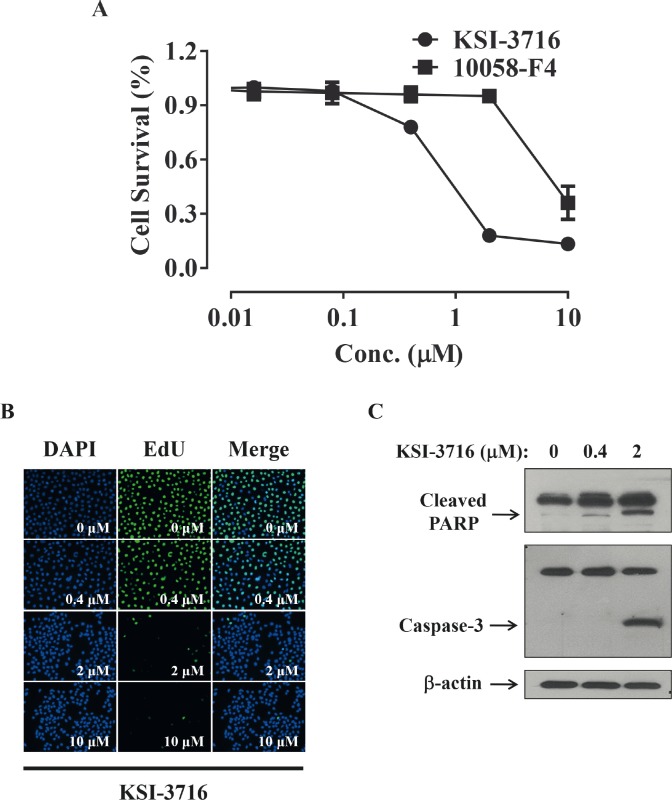
*In vitro* cell death assay for KU19-19/GEM cells in the presence of KSI-3716 (**A**) KU19-19/GEM cells (3 × 10^3^ cells/well) were plated in 96-well plates and cell proliferation was measured after treatment with 0–10 μM KSI-3716 or 10058-F4. (**B**) KU19-19/GEM cells (1 × 10^4^ cells/well) were seeded in 96-well black plates and treated with 0–10 (μM KSI-3716. After 48 hrs, intercalated EdU was detected as above. (**C**) KU19-19/GEM cells (5 × 10^5^) were plated in 100 mm dishes and harvested 48 hrs after incubation with 0–2 μM KSI-3716. PARP and caspase-3 cleavage fragments were detected by western blotting with 50 μg of total cell lysate per lane.

Since *c-myc* gene amplification is found in up to 30% of bladder cancer patients, immunohistochemical staining of a tissue microarray was performed to evaluate c-Myc expression in bladder tumors. Three different staining patterns were identified in the bladder tumor samples (Supplementary Figure 1A) and are summarized in Supplementary Table 1. Furthermore, all the bladder cell lines tested also showed high levels of *c-myc* transcripts, as determined by PCR (Supplementary Figure 1B). Since cancer cells are highly likely to modulate their protein expression in response to a cytotoxic milieu, c-Myc expression was investigated in the presence of gemcitabine. Cells were exposed to 10 μM gemcitabine for 24 and 48 hrs, and c-Myc protein was detected by immunoblot analysis (Figure 4). Interestingly, KU19-19/GEM cells expressed higher levels of c-Myc protein (by 30%) in the presence of gemcitabine. These results suggest that c-Myc may contribute to cell survival or gemcitabine resistance, and c-Myc inhibitors could be effective agents for inhibition of cell proliferation. We previously demonstrated that intravesical instillation of the c-Myc inhibitor KSI-3716 markedly inhibits tumor growth in a mouse orthotopic bladder cancer model [25]. Thus, we investigated whether KSI-3716 could also inhibit the proliferation of KU19-19/GEM cells. KSI-3716 inhibited cell survival by 85% at 2 μM in the KU19-19/GEM cell line and was much more cytotoxic than the c-Myc inhibitor 10058-F4 (Figure 5A). Cell proliferation in the presence of KSI-3716 was determined using an EdU incorporation assay, which confirmed a marked inhibition of DNA synthesis at 2 μM KSI-3716 (Figure 5B). However, when cells were treated with another c-Myc inhibitor 10058-F4 at doses of up to 10 μM, no inhibition of cell proliferation was observed (data not shown). Poly (ADP-ribose) polymerase (PARP) and caspase-3 cleavage were detected by western blotting, and indicated induction of apoptosis (Figure 5C), consistent with the inhibition of cell proliferation (above).

### Augmentation of therapeutic potency by sequential addition of gemcitabine and KSI-3716

**Figure 6:**
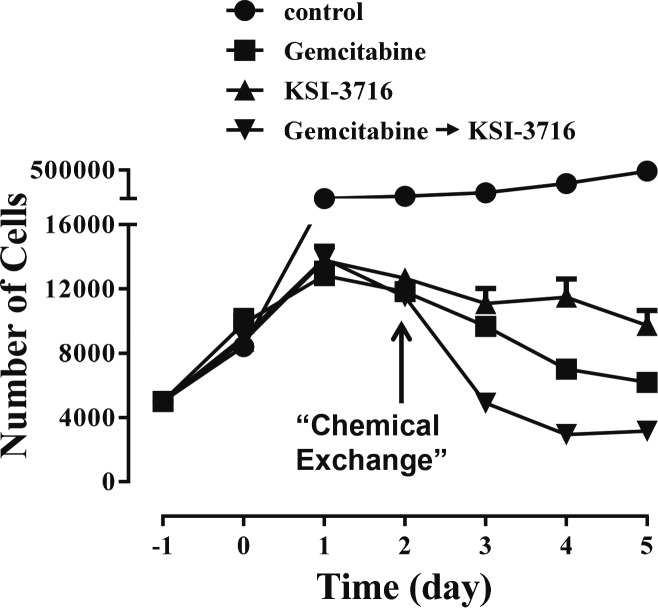
Sequential addition of gemcitabine and KSI-3716 enhance the anti-cancer potency (**A**) KU19-19 cells (5 × 10^3^ cells/well) were plated in 24-well plates. After 24 hrs, cells were treated with 1 μM KSI-3716 or 0.1 μM gemcitabine for 2 days. Culture media were then replaced with fresh media containing either KSI-3716 or gemcitabine. Cell number was measured daily using an automatic cell counter after trypsinizing cells in replicated plates.

The c-Myc inhibitor KSI-3716 induced apoptosis and blocked KU19-19/GEM cell proliferation, suggesting that KSI-3716 could be an effective chemotherapeutic agent, particularly when combined with gemcitabine. We therefore explored how to maximize the anti-cancer actions of both drugs, and tested KU19-19 cell proliferation to determine if cytotoxic potency was increased when both drugs were combined. KU19-19 cells (prior to the development of gemcitabine resistance) were administered with both drugs simultaneously, and cell proliferation was measured. However, the effects of co-administration were no different to those of the c-Myc inhibitor alone (Supplementary Figure 2), Next, we exposed KU19-19 cells to 0.1 μM gemcitabine for 2 days and then added KSI-3716 to the surviving gemcitabine-resistant cells after removal of gemcitabine (Figure 6). Sequential addition of each drug inhibited cell proliferation to a greater extent than each drug alone. These results suggest that KSI-3716 could be used upon termination of gemcitabine treatment to synergistically inhibit cancer proliferation and prevent tumor recurrence by gemcitabine-resistant cells.

## DISCUSSION

Transurethral resection followed by intravesical immuno- or chemotherapy is the standard therapy to prevent recurrence and progression in patients with intermediate to high risk NMIBC. The most commonly used drug for intravesical therapy is the BCG agent. Although widely used, there can be significant adverse reactions to BCG and a high risk of treatment failure. When patients do not respond to BCG or recur eventually, a radical cystectomy remains standard treatment. However, many patients are medically unfit and refuse this operation because radical cystectomies are also associated with significant morbidity and reduced quality of life [26]. Urothelical carcinoma is characterized by chemo-sensitivity and best responses are seen using multidrug platinum-based regimens in metastatic or advanced bladder cancer. Combining different agents can decrease chemotherapy resistance and is often successfully used to improve response rates to systemic therapy. This strategy has been tested in NMIBC. However, studies have not identified any clear benefit to doing so in intravesical therapy.

During the last several decades, a number of chemotherapeutic agents have been introduced and protocols established to reflect different types of cancer and patient physiology. However, drug resistance is a major problem. Furthermore, patients face the emergence of multidrug resistance (MDR), defined as resistance to structurally or functionally unrelated drugs. There are two types of MDR; intrinsic and acquired [27, 28], For patients with gemcitabine refractory cancer, it is sometimes assumed that they are less likely to receive benefits from other conventional chemotherapies. Here, we demonstrated that the c-Myc inhibitor KSI-3716 was cytotoxic to gemcitabine-resistant bladder cancer cells, suggesting that c-Myc inhibitors could be a viable treatment option when MDR is involved. In addition to gemcitabine-resistant cells, KSI-3716 also effectively induced cell death in paclitaxel-resistant KU19-19 cells, as shown in Supplementary Figure 3. Since the transcription factor, c-Myc plays a critical role in cancer initiation and progression [29], inhibition of c-Myc expression or activity could also be an effective therapeutic strategy for MDR, including gemcitabine resistance.

Deregulation of c-Myc is critical for the development of many human cancers [22]. New evidence has uncovered a previously unknown mechanism whereby increased abundance of c-Myc can promote PARP-dependent DNA repair pathways and induce relative chemo-resistance [21]. In a previous report, we demonstrated that KSI-3716, which inhibits c-Myc/MAX/DNA complex formation, can be instilled into the bladder to effectively suppress tumor growth without noticeable systemic toxicity, demonstrating a novel use for c-Myc inhibitors. Here, we established a gemcitabine-resistant cell line, termed KU191-9/GEM. Interestingly, KU19-19/GEM cells were selectively resistant to gemcitabine, but not to paclitaxel, doxorubicin, and mitomycin C. These data suggest that a combination of gemcitabine plus any of the above may be useful treatment for gemcitabine-resistant cells.

Interestingly, simultaneous addition of KSI-3716 and gemcitabine did not increase cytotoxicity compared with c-Myc inhibitor alone (Supplementary Figure 2), suggesting that the cell survival response to one drug blocks the cytotoxic action of the other drug. However, sequential addition augmented cytotoxic anti-cancer effects in KU19-19 cells. Drug resistance develops through a number of mechanisms [27, 28], specifically: 1) changes in tumor structure, such as vasculature leakage and tumor hypoxia; and 2) modulation of gene expression, including oncogenes, DNA repair genes, and changes in sensitivity to growth factors and nutrients. Therefore, it is likely that gemcitabine-resistant KU19-19 cells temporarily up-regulate genes required for drug resistance, such as anti-apoptotic genes, so that sequential addition is more effective than simultaneous treatment.

Cancer stem cells are recognized as the underlying cause of tumor initiation, recurrence, and growth [30, 31]. In addition, the main reason for drug resistance is the incomplete removal of cancer stem cells [32] and MDR cells have stem-like properties [33]. As shown in Figure 3D, the phenotype of the KU19-19/GEM cells were quite different from that of the KU19-19 cells and considered undergoing epithelial-mesenchymal transition (EMT). We investigated gene expression profiles in KU19-19 and KU19-19/GEM cells using microarrays, and focused on cancer stem cell-related genes (data not shown). Notably, KU19-19/GEM cells overexpressed stem cell-related genes, including CXCR4, Sox9 and Sox2. These data suggest that KU19-19/GEM could have a cancer stem cell-like phenotype that mediates gemcitabine resistance.

For clinical applications, although c-Myc inhibitors are generally considered to be non-tolerable due to systemic toxicity, our previous results and the current study demonstrate that KSI-3716 can be used to treat tumors intravesically regardless of gemcitabine resistance. Sequential treatment with KSI-3716 therefore represents a promising new strategy for the clinical applications of gemcitabine.

## MATERIALS AND METHODS

### Cell lines and cell culture

Human (T24 and 253J) and mouse (MBT-2) bladder cancer cell lines were maintained in RPMI 1640 supplemented with 10% fetal bovine serum (FBS) and 1% penicillin/streptomycin (Invitrogen, Carlsbad, CA). The human bladder cancer cell line KU19-19 was donated by Dr. Ozu (Tokyo Medical University) and maintained in minimal essential medium (MEM) supplemented with 10% FBS and 1% penicillin/streptomycin.

### Cell cycle analysis and detection of apoptosis and cytotoxicity

To quantify cell proliferation, a cell proliferation assay (CellTiter-Glo, Promega, Madison, WI) was performed using the standard protocol, with some modifications. Briefly, 3 × 10^3^ cells were seeded in 96-well plates, 2 days later cells were exposed to chemotherapeutic agents, and cell proliferation was measured the next day. All experiments were performed in quintuple. For cell cycle analysis, cells were harvested, washed, fixed with ice-cold 70% ethanol, and stained with 50 μg/ml propidium iodide (PI) in the presence of 100 U RNase A for 30 min at 37°C. For flow cytometer analysis, at least 10,000 events were acquired and results were analyzed with CellQuest software (Becton Dickinson, San Diego, CA). EdU cell proliferation assays were performed using the Click-iT EdU assay kit (Invitrogen). Cells were seeded into 96-well black plates and incubated at 37°C for 1 day. Chemotherapeutic agents of interest were added to each well at a final concentration of 0 to 10 μM. Following incubation for 6 hrs, 10 μM of EdU was added to the cell culture plate for 18 hrs, and cells were fixed with formaldehyde. The fixed cells were permeabilized with 0.1%) Triton X-100 in phosphate-buffered saline (PBS). Finally, intercalated EdU was detected with 5 μM Alexa Fluor 488-conjugated azide and visualized by fluorescence microscopy.

### Western blotting and histological analysis

For western blot analyses, cells were lysed in RIPA buffer containing protease inhibitors (Sigma-Aldrich, St. Lois, MO). Proteins (20–50 μg) were resolved by SDS-PAGE and transferred to a PVDF membrane. Membranes were probed with antibodies against c-Myc, PARP, or caspase 3 (all from Cell Signaling, Danvers, MA), and the subsequent corresponding secondary antibody (Jackson Immuno Research Laboratories, West Grove, MA) was detected using an Enhanced Chemiluminescence (ECL) Plus kit (Thermo Fisher Scientific, Waltham, MA). For histological analyses, tumor sections of 5–10 μm were affixed to slides, de-waxed with ethanol, and stained with hematoxylin and eosin.

### Animal studies

All animal experiments in this study were performed in accordance with the Guidelines for the Care and Use of Laboratory Animals of the National Cancer Center of the Republic of Korea (approval number, NCC-11-122). Subcutaneous xenografts were formed by injection of 2 × 10^6^ KU19-19 or KU19-19/GEM cells into the flank of BALB/c nude mice. When tumors were palpable, mice were treated with gemcitabine (by intraperitoneal injection, 2 mg/kg) twice a week for 3 weeks. Tumors were measured twice a week and were harvested for histological analyses at the end of the study.

### TUNEL assay

The TUNEL assay was performed using a one-step TUNEL kit (Millipore, Billerica, MA) to label free 3′OH DNA termini in KU19-19 tumors. For the detection of apoptotic cells, tumor sections were mounted on slides, rinsed with PBS, and then permeabilized with 0.1% Triton X-100 for 2 min on ice. The TUNEL assay was then performed according to the manufacturer's instructions. Cyanine 3-labeled TUNEL-positive cells were imaged under a fluorescence microscope. Cells labeled with red fluorescence were considered apoptotic.

## Supplementary Figures and Tables


