# Supplementary material for: Antitumor activity of the c-Myc inhibitor KSI-3716 in gemcitabine-resistant bladder cancer
Source: Oncotarget. 2014 Jan 16;5(2):326–37. doi: 10.18632/oncotarget.1545 (PMC3964210; doi:10.18632/oncotarget.1545)
Supplement: Supplementary file 1 [file oncotarget-05-326-s001.pdf]

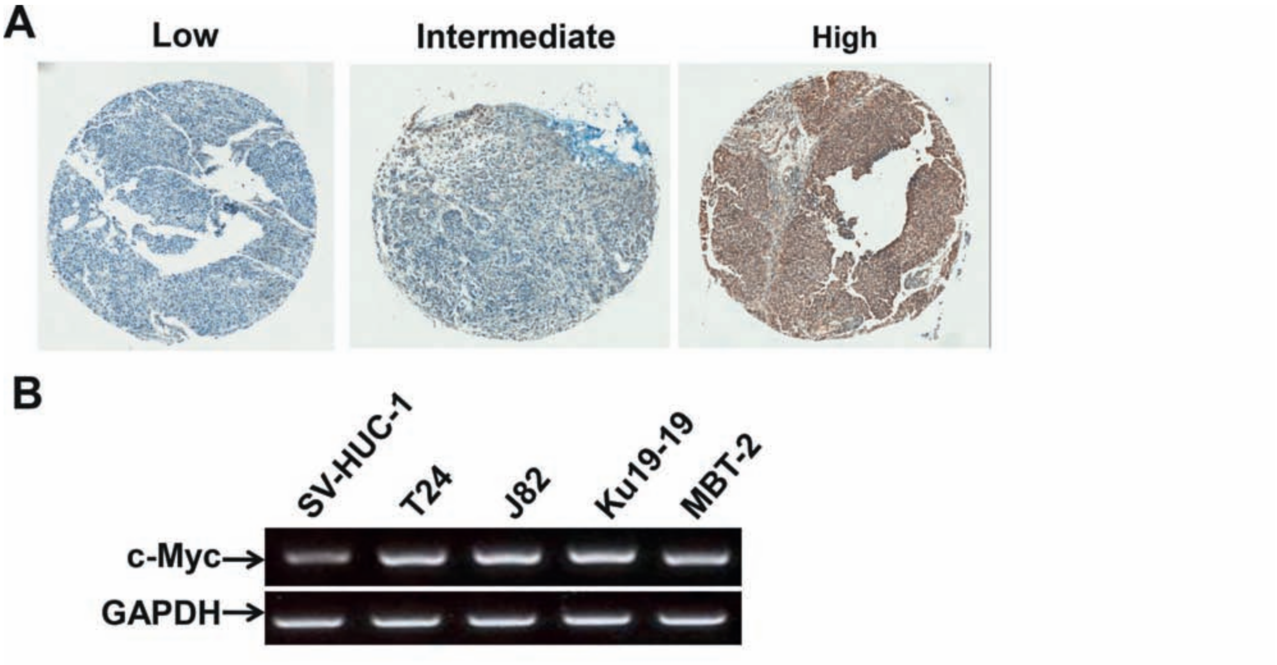

Supplementary Figure S1. c-Myc is highly expressed in all bladder cancer cells and in some bladder tumor samples on tissue microarray (TMA). (**A**) C-Myc expression in patient samples prepared in the form of a bladder cancer TMA was analyzed by immunohistochemistry using an anti-c-Myc antibody. On the basis of staining results, patients were grouped into high, intermediatelow, and low negative Expression of c-Myc, compared with their normal tissue. (**B**) Total RNA was prepared from each sample and 1 μg was reverse-transcribed. PCR was performed to detect *c-myc* transcripts in the synthesized cDNA.

Supplementary Table S1: Patient demographics and c-MYC expression in bladder cancer samples

	Number (%)
No. of patients	40
Age (years)	58.9 ± 11.1
Sex	
Male	29 (72.5%)
Female	11 (27.5%)
Stage	
NMIBC	15 (37.5%)
MIBC	25 (62.5%)
Grade	
1	8 (20.0%)
2	27 (67.5%)
3	5 (12.5%)
c-MYC expression	
Negative	15 (37.5%)
Low	17 (42.5%)
High	8 (20.0%)

NNIBC: non-muscle invasive bladder cancer MIBC: muscle invasive bladder cancer MBC: metastatic bladder cancer.

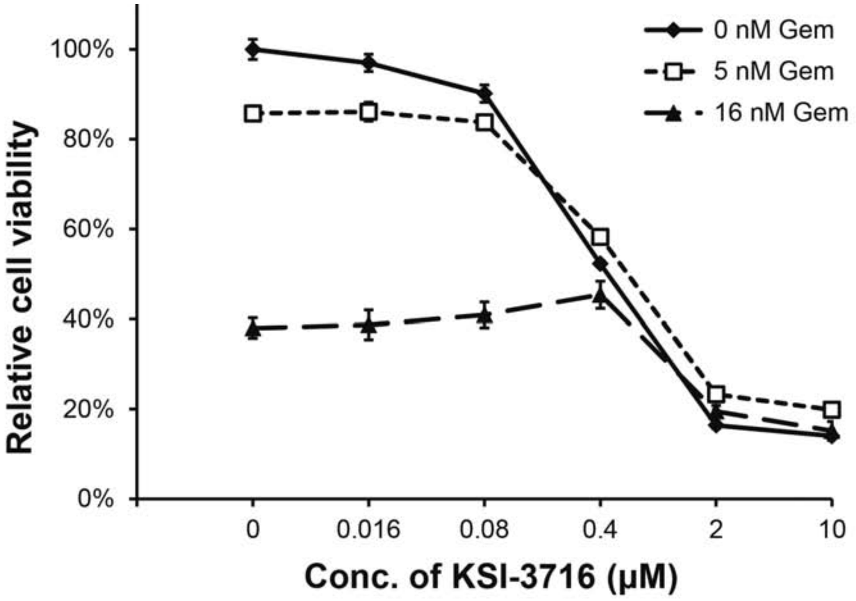

Supplementary Figure S2. Simultaneous addition of KSI-3716 and gemcitabine did not increase cytotoxicity compared with gemcitabine c-Myc inhibitor alone. KU19-19 cells were exposed to 0 – 10 μM of KSI-3716 with or without gemcitabine. Regardless of gemcitabine concentration, there was no additional effect in the cytotoxicity of KSI-3716.

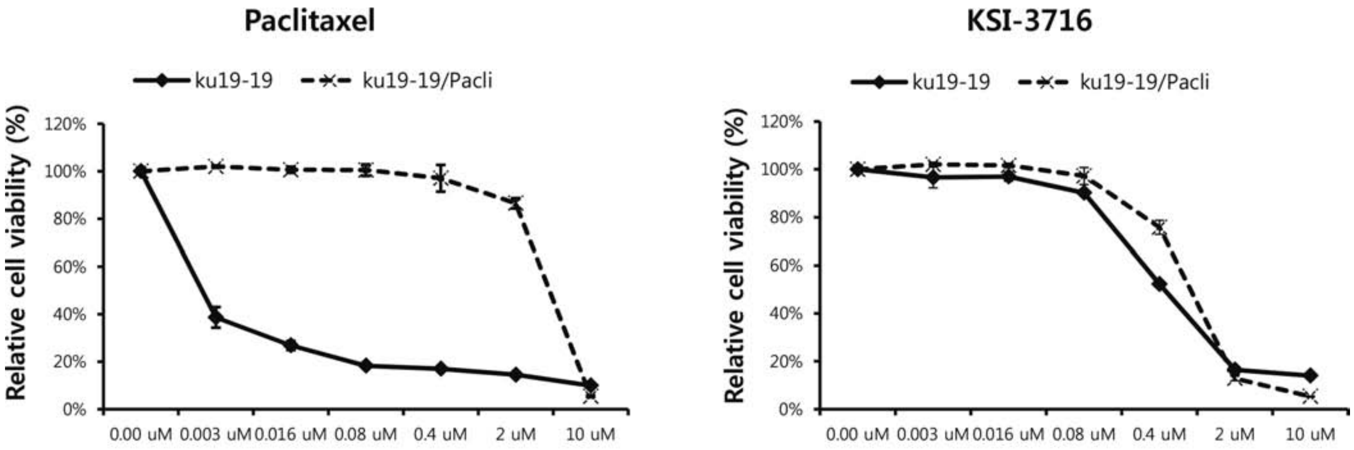

Supplementary Figure S3. c-Myc inhibitor KSI-3716 is effectively kills paclitaxel-resistant KU19-19 cells. We also established paclitaxel-resistant KU19-19 cell (termed KU19-19/Pacli) and its IC_{50} value was 10,000 fold higher than parental paclitaxel-sensitive KU19-19 cell. Interestingly, there was no difference between two cells' sensitivity to KSI-3716.
